# Successful Management of Lumbosacral Spondylodiscitis Using All-Posterior Four-Rod Spinopelvic Fixation Without Anterior Reconstruction: A Case Report and Literature Review

**DOI:** 10.7759/cureus.111265

**Published:** 2026-06-21

**Authors:** Rishab Kafley, Siddharth s Sethy, Aditya P Panda, Srikant Konchada, Shankar Acharya

**Affiliations:** 1 Orthopedics, Kalinga Institute of Medical Sciences, Bhubaneswar, IND

**Keywords:** all-posterior approach, lumbo-pelvic fixation, lumbosacral junction, spondylodiscitis, tuberculosis

## Abstract

Spondylodiscitis of the lumbosacral spine is uncommon and presents unique diagnostic and therapeutic challenges. The compromised stability of this junction due to infection often necessitates surgical intervention. Anterior column reconstruction usually becomes necessary given the inherent shear force at this junctional area. Our case represents a unique management strategy without anterior column reconstruction; three-column stability was achieved by modifying fixation with a four-rod technique instead of the standard two rods. We report a 49-year-old female with insidious-onset low back pain, bilateral leg tingling, and constitutional symptoms. Imaging revealed destruction from L4 to S1 with sacral ala involvement and right sacroiliac joint changes, consistent with tuberculous spondylodiscitis. Laboratory markers were elevated, and the Tuberculosis Spine Instability Score (TSIS) was 12. The patient was empirically started on anti-tubercular therapy (ATT) and underwent an all-posterior approach with decompression, debridement, and lumbo-pelvic fixation using a four-rod construct. Postoperatively, she was mobilized early, discharged on day 7, and continued 18 months of ATT. At two-year follow-up, she showed solid fusion and an intact construct. She marked functional recovery, with the Oswestry Disability Index improving from 90% to 10% and the Visual Analog Scale score from 9/10 to 0. Posterior-only lumbo-pelvic fixation with a four-rod construct provides a safe and effective solution for extensive lumbosacral tuberculous spondylodiscitis with sacral extension. In endemic regions, empirical ATT based on clinicoradiological suspicion is justified even when microbiological confirmation is lacking.

## Introduction

Spondylodiscitis accounts for approximately 2%-5% of all osteomyelitis cases and poses significant diagnostic and therapeutic challenges, particularly when it involves the lumbosacral junction [1]. It is characterized by infection of the intervertebral disc and adjacent vertebral endplates, resulting in progressive destruction of spinal structures, pain, deformity, and, in advanced cases, neurological compromise. Delayed diagnosis is common because of its often indolent clinical presentation, leading to substantial morbidity and functional disability [2]. Infection at the lumbosacral junction is especially challenging because this region serves as a biomechanical transition zone between the mobile lumbar spine and the rigid sacropelvic complex, transmitting considerable axial and shear forces during daily activities. Surgical management is indicated in patients with neurological deficits, significant vertebral destruction, progressive deformity, abscess formation, or mechanical instability [3].

Among the various etiologies, tuberculosis (TB) remains a leading cause of spondylodiscitis in endemic regions such as India and is one of the most common forms of extrapulmonary TB [4]. Anterior column destruction at the lumbosacral level can rapidly result in segmental instability and compromise overall spinal alignment. Tuberculous spondylodiscitis typically presents insidiously with chronic back pain, constitutional symptoms, paraspinal abscess formation, and late neurological deficits due to delayed presentation [2,4,5,6].

While anterior decompression and reconstruction have traditionally been considered the standard surgical approach because of direct access to the diseased anterior column, recent advances in spinal instrumentation have established posterior-only approaches as a viable and less invasive alternative [3]. Isolated involvement of the lumbosacral spine is relatively rare, making diagnosis and management particularly difficult because of the complex anatomy and high mechanical demands of this region [4,7]. Posterior decompression and fixation offer several advantages, including reduced tissue dissection, shorter operative time, lower perioperative morbidity, earlier mobilization, faster rehabilitation, and shorter hospital stay compared with anterior approaches [3,8]. However, in cases with marked anterior column destruction, conventional two-rod lumbosacral fixation constructs may not provide sufficient biomechanical stability. We present a unique case in which, despite significant anterior column destruction secondary to lumbosacral tuberculous spondylodiscitis, robust three-column stability was successfully achieved by modifying standard lumbosacral fixation using a four-rod construct instead of the conventional two-rod technique.

## Case presentation

A 49-year-old female with no known comorbidities presented with a one-month history of insidious-onset low back pain and bilateral leg tingling. Constitutional signs, including low-grade fever and weight loss, accompanied the symptoms. Initial management with analgesics and rest provided minimal relief. The pain progressively worsened, leading to significant functional impairment that required the use of a cane for ambulation and limited her to only basic activities of daily living. Throughout the disease progression, her bowel and bladder functions remained intact. Physical examination revealed marked tenderness over the lumbosacral junction, with severe pain elicited on minimal movement. There was no palpable deformity or swelling. The neurological assessment was unremarkable, with intact motor power (5/5 in all myotomes), normal sensation, and intact deep tendon reflexes. Preoperative computed tomography imaging (Figure 1) confirmed the involvement of L4 to S1 vertebrae with cystic destruction, right-sided sacral alar involvement, and endplate erosion.

**Figure 1:**
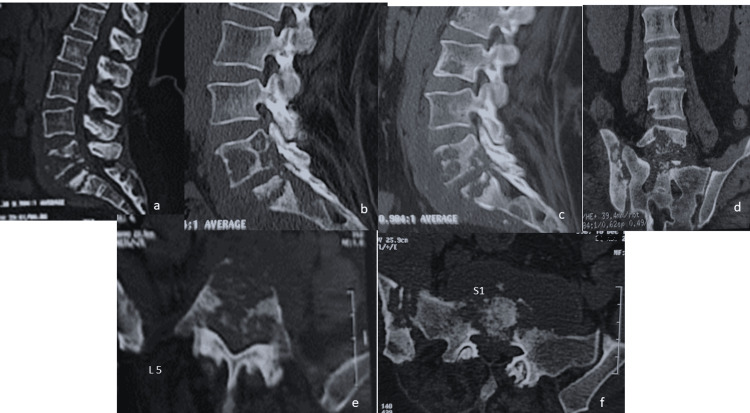
Computed tomography showing an osteolytic lesion involving the L5–S1 vertebrae in the midsagittal view (a), with intact facet joints in the parasagittal sections (b, c), along with involvement of the right sacroiliac joint in the coronal view (d), and destruction of the L5 and S1 vertebral bodies in the axial sections (e, f).

Magnetic resonance imaging (Figure 2) revealed evidence of altered marrow signal intensity, characterized by T1 hypointensity and T2/short tau inversion recovery (STIR) hyperintensity, involving the left half of the L2-L3 vertebral bodies and the intervertebral disc, causing a reduction in disc space height.

**Figure 2:**
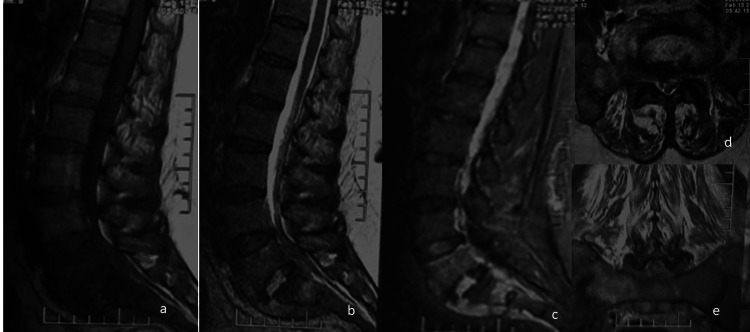
MRI showing altered marrow signal intensity in the form of T1 hypointensity (a) and T2/short tau inversion recovery (STIR) hyperintensity (b, c) in sagittal sections involving the L5–S1 vertebral bodies, with a hyperintense L5–S1 disc space and prevertebral collection. T2-weighted axial section (d) showing hyperintense paravertebral collections at the S1 level, along with right sacroiliac joint involvement, also evident in the coronal section (e).

Mild paravertebral soft-tissue thickening showing STIR hyperintensity was also noted along the left lateral margins of L2-L3; similar changes were also noted in the right half of the L4 vertebral body. There was evidence of near destruction of the L5-S1 vertebral junction, with collapse of the disc space and adjacent paravertebral soft-tissue thickening. The right sacroiliac joint showed surrounding marrow edema and erosions involving the adjacent iliac bone and right sacral ala, confirming infective spondylodiscitis at the same levels with paravertebral soft-tissue involvement.

Her laboratory investigations showed elevated erythrocyte sedimentation rate (ESR) (92 mm/hr), C-reactive protein (CRP) (90 mg/L), and total leukocyte count (TLC) (10,080/mm³). Preoperatively, the Oswestry Disability Index (ODI) score [9] was 90%, the Visual Analog Scale (VAS) score [10] was 9/10, and the Tuberculosis Spine Instability Score (TSIS) [11] was 12.

She was empirically started on anti-tubercular therapy (ATT) 15 days before surgery based on clinicoradiological suspicion and the endemicity of tuberculosis in India. Surgery was performed in view of spinal instability. The procedure was carried out under general anesthesia in the prone position through a posterior midline incision, and the L3-S2 vertebrae were exposed. Pedicle screws were inserted in L3-Iliac and L4-S2. Decompression was performed with L5-S1 laminotomy and discectomy (Figure 3).

**Figure 3:**
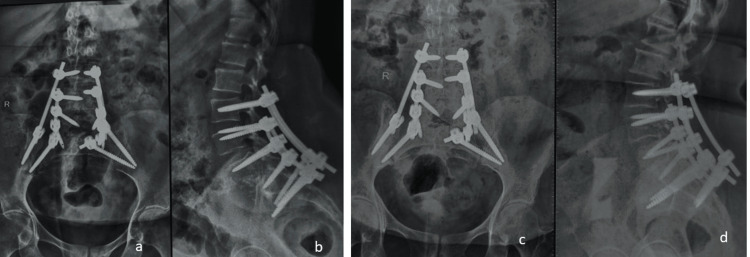
Immediate postoperative radiograph showing lumbopelvic fixation with a four-rod construct in the anteroposterior (a) and lateral (b) views. Follow-up radiograph at 2 years showing intact lumbopelvic alignment with the implant in situ in the anteroposterior (c) and lateral (d) views.

Debridement was done thoroughly to remove devitalized tissue, and all-posterior lumbopelvic fixation with a bone graft was performed using a four-rod construct. Intraoperative tissue samples were sent for histopathological and microbiological examination.

Postoperative recovery was uneventful. Histopathology revealed moderate inflammatory cells, with no granuloma or malignancy. Cultures showed no growth of organisms, and the cartridge-based nucleic acid amplification test (CBNAAT) was negative. She received injectable cefotaxime + sulbactam 1.5 g twice daily and injection gentamicin 80 mg once daily for five days, along with a full course (18 months) of ATT as per the National Tuberculosis Elimination Program (NTEP) [12]. The immediate postoperative period was uneventful, and she was discharged on postoperative day 7.

The patient was followed up regularly every month for the first three months and then on a three-monthly basis for the first year and biannually thereafter. The ODI scores at one month, three months, six months, one year, and two years postoperatively were 48%, 30%, 21%, 16%, and 10%, respectively. The VAS scores at one month, three months, six months, one year, and two years postoperatively were 6, 4, 1, 0, and 0, respectively. Currently, two years postoperatively, the patient is an independent ambulator performing her activities of daily living. Follow-up radiological investigation showed good fusion (Figure 4) and maintained alignment.

**Figure 4:**
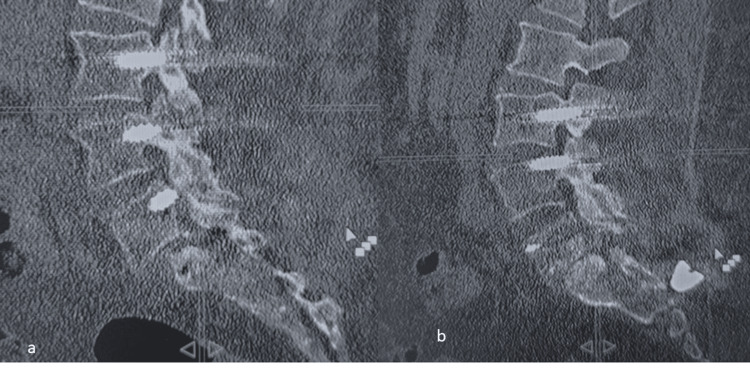
Non-contrast computed tomography (NCCT) showing bony fusion of the facet joints in parasagittal sections (a, b) at two-year follow-up.

MRI at two years was suggestive of healing without any signs of disease relapse/recurrence (Figure 5).

**Figure 5:**
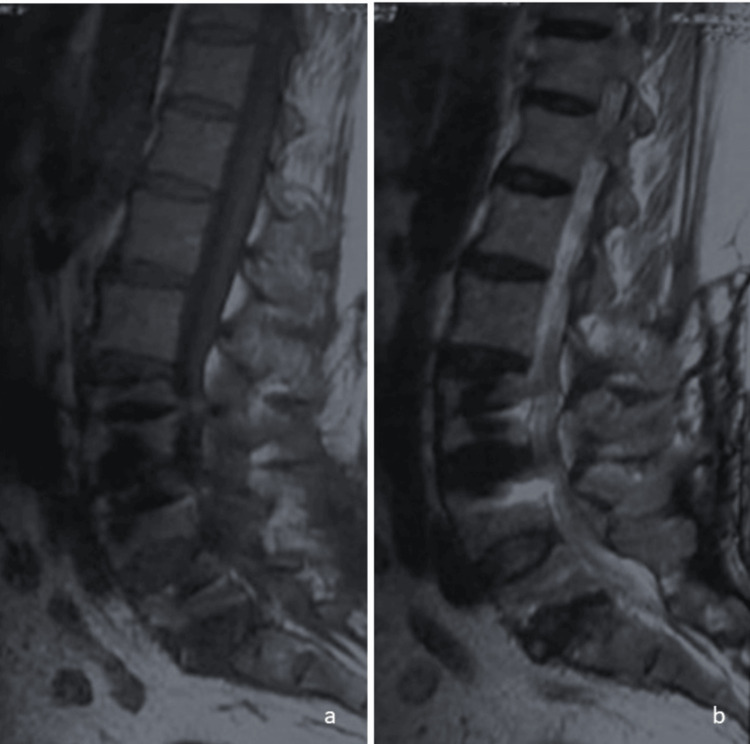
MRI showing a healed lesion with hypo- to isointense signal intensity in T1 (a) and T2 (b) sections.

## Discussion

The lumbosacral region acts as a pivot between the mobile lumbar lordotic segment and the fixed sacral kyphotic segment. This, in turn, creates substantial shear forces that must be borne by the junctional segment. Any compromise of structural integrity can jeopardize stability, leading to pain and/or neurological compromise. Surgical intervention is deemed necessary in the presence of spinal instability [11], along with antitubercular treatment. A literature review of lumbosacral tuberculosis is presented in Table 1.

**Table 1:** Literature review of case reports on lumbosacral tuberculosis.

Serial no.	Author	Year	Demography	Duration	Medical management	Surgical procedure	Fusion follow-up
1.	Shah et al. [13]	2012	24 years/Female	Not applicable	Empirical Category I anti-tuberculosis treatment.	Lumbopelvic instrumented fixation and S1 body reconstruction with structural allograft	Symptom-free at two years
2.	Uvaraj et al. [14]	2014	21 years/Female	6 months	Category I anti-tuberculosis treatment.	Posterior instrumentation T10-11, 12-iliac pedicle screws, and anterior fibular strut grafting	Achieved at 61 months
3.	Agrawal et al. [15]	2015	45 years/Male	1.5 months	Category I anti-tuberculosis treatment.	Surgical decompression, spinal fixation, and evacuation of the abscess	Improved motor and sensory deficits and completely pain-free.
4.	Rathod et al. [16]	2020	42 years/Female	3 months	Category II anti-tuberculosis treatment.	All posterior decompression with instrumentation from D10 to S2, with a reconstruction of anterior vertebral bodies with an expandable cage	Walk without support and do activities of daily living and household work at 3.5 years
5.	Arora et al. [17]	2023	Late 20s/Male	1.5 years	Over-the-counter analgesics, Category I anti-tuberculosis treatment.	None	Transferred under directly observed treatment, short-course to ensure compliance and a cure at one year
6.	Our study	2026	49 years/Female	1 month	Category I anti-tuberculosis treatment.	Posterior decompressive L5-S1 laminotomy with instrumentation L3-Iliac and L4-S2 (four-rod construct)	Fusion at two years, symptom-free

Although the patient in the current discussion was neurologically sound, multilevel gross destruction of the junctional segment (TSIS-12) necessitated management with surgical stabilization in our case.

Involvement of the sacrum in lumbosacral spondylodiscitis is rare and limits fixation options. Cases reported by Shah et al. [13], Uvaraj et al. [14], and Rathod et al. [16] included in our review required anterior column reconstruction in view of loss of structural integrity. Traditional approaches often favor anterior debridement followed by posterior stabilization. However, anterior approaches carry a higher risk, especially at the lumbosacral junction, due to proximity to iliac vessels, bowel, and ureters [18]. The surgical morbidity also increases with staged procedures in terms of increased surgical time, blood loss, and hospital stay. The posterior-only approach, particularly with lumbo-pelvic fixation using iliac screws, allows for indirect decompression and improved stability and avoids the morbidity of anterior exposure [19]. Anterior column reconstruction was not required in this case, with good index screw purchase and well-maintained global alignment [20]. Furthermore, the use of a four-rod construct has shown mechanical advantage, especially in long-segment stabilization of compromised bone, as supported by the biomechanical rigidity of iliac fixation constructs [21,22]. The follow-up at two years shows good sound fusion, and the patient is mobile without any aid. Several studies have also validated the advantage of the posterior-only technique in addressing the anterior disease of spondylodiscitis [23,24].

The occurrence of negative cultures, despite clear clinical and radiological signs of infection, is not uncommon. This is particularly true in cases of partially treated tuberculous infections or low-grade pyogenic spondylodiscitis [25]. TB is a treatable condition and is associated with severe adverse outcomes if left unmanaged. In the presence of a high index of diagnostic clinicopathological suspicion [26], empirical treatment with ATT is justified [27]. In our patient, the decision to commence empirical ATT was based on this principle and supported by the failure to isolate any pyogenic organisms from culture. This case underscores the critical importance of maintaining a high index of suspicion for TB in endemic zones, even in the absence of microbiological confirmation.

Our literature review of lumbosacral TB suggests that, depending on the stage, early disease without instability or neurological compromise can be managed with medical ATT [15]. The patients with instability or deficits who need decompression and fixation along with ATT give optimal results in terms of fusion, pain relief, and functional recovery [13,14,16]. Patients with advanced disease and involvement of multiple segments requiring anterior column reconstruction benefit from circumferential reconstruction [14,16]. Nevertheless, posterior-only fixation, as demonstrated in our case, can achieve comparable functional outcomes and fusion rates.

## Conclusions

In conclusion, an all-posterior approach with lumbopelvic fixation using a four-rod construct represents a safe, effective, and single-stage surgical solution for extensive spondylodiscitis of the lumbosacral spine with sacral extension. Furthermore, this case reinforces the principle that in endemic regions, initiating empirical ATT is a crucial component of management, justified by a high index of clinicoradiological suspicion, even in the absence of microbiological confirmation.
